# Wind estimation around the shipwreck of Oriental Star based on field damage surveys and radar observations

**DOI:** 10.1007/s11434-016-1005-2

**Published:** 2016-02-02

**Authors:** Zhiyong Meng, Dan Yao, Lanqiang Bai, Yongguang Zheng, Ming Xue, Xiaoling Zhang, Kun Zhao, Fuyou Tian, Mingjun Wang

**Affiliations:** Laboratory for Climate and Ocean–Atmosphere Studies, Department of Atmospheric and Oceanic Sciences, School of Physics, Peking University, Beijing, 100871 China; National Meteorological Center, Beijing, 100081 China; School of Atmospheric Sciences, Nanjing University, Nanjing, 210023 China

**Keywords:** Squall line, Bow echo, Microburst, Damage survey, Drone, Tornado, 飑线, 弓形回波, 微下击暴流, 灾害调研, 无人机, 龙卷

## Abstract

Based on observational analyses and on-site ground and aerial damage surveys, this work aims to reveal the weather phenomena—especially the wind situation—when Oriental Star capsized in the Yangtze River on June 1, 2015. Results demonstrate that the cruise ship capsized when it encountered strong winds at speeds of at least 31 m s^−1^ near the apex of a bow echo embedded in a squall line. As suggested by the fallen trees within a 2-km radius around the wreck location, such strong winds were likely caused by microburst straight-line wind and/or embedded small vortices, rather than tornadoes.

**Electronic supplementary material:**

The online version of this article (doi:10.1007/s11434-016-1005-2) contains supplementary material, which is available to authorized users.

## 摘要

2015年6月1日,长江流域湖北监利航段发生了“东方之星”号客轮特大倾覆事故,造成442人遇难。本文基于雷达观测和地面及无人机航拍灾害调研,揭示了事故时段的天气现象,尤其是风力情况。研究表明,客轮在倾覆时处于一条飑线的弓形回波前缘,遭遇了至少31 m/s的强风。客轮倾覆位置2 km范围内的树木倒伏特征表明,该强风可能是由微下击暴流里的直线大风或其中的小尺度涡旋造成,而并非龙卷。

## Introduction

**Fig. 1:**
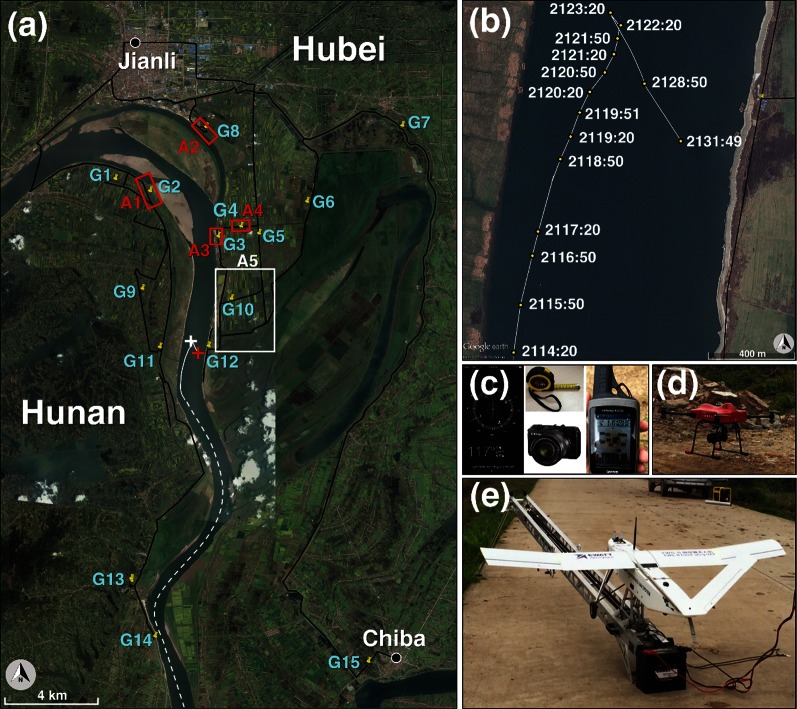
Damage survey information along with the sailing trace of Oriental Star on a satellite image obtained from Google Earth. **a** Routes (black lines) and the sites (yellow pins, marked with a letter and a number) of the damage survey. The four red boxes and one large white box at A1–A5 denote the areas surveyed by drones, while G1–G15 were surveyed by the authors on the ground. The black dots denote the surface weather stations at Jianli and Chiba. The red (white) cross denotes the wreck location (northernmost location) of Oriental Star. Also given in (**a**) is the sailing trace of the ship during its last 21 min (white solid line), based on the real-time AIS records of the China Portage Network, which is partly enlarged in (**b**) and denoted in LST (shipwreck occurred at ~2131 LST), and a schematic earlier trace (white dashed line) not strictly based on the records. **c** Digital compass, ruler, camera, and mobile GPS used in the damage survey. **d** Vertical takeoff and landing drone used to survey A1–A4 via videos. **e** Fixed-wing drone used to survey A5 via continuous photographs. North is indicated by an arrow in a gray-shaded circle in (**a**, **b**)

Oriental Star, a cruise ship on its way to Chongqing from Nanjing with 454 people on board, capsized on the Yangtze River in Jianli County, Hubei Province, China, at about 2131 LST (local standard time; LST = UTC + 0800) on June 1, 2015, leaving 442 fatalities (Fig. 1a, b). This disaster happened when the ship encountered a severe thunderstorm. The objective of this paper is to reveal what kind of weather phenomena occurred and how strong the wind was around the wreck location.

According to the real-time records from the Automatic Identification System (AIS) (Fig. 1b) of the China Portage Network, Oriental Star started to show a rightward shift at 2120:20 LST (the number after the colon denotes seconds) while it was sailing north–northeastward upstream near the left shore of the Yangtze River. It then took a sharp turn to the northwest at 2122:20 LST. One minute later at 2123:20 LST, the ship started to retreat backward to the southeast and capsized at about 2131 LST. The last AIS signal was received at 2131:49 LST. As recalled by the crewmembers, the ship was hit twice by extremely strong winds: The first time was around 2123 LST when the ship reached the northernmost point and started to retreat and the second time was at about 2126 LST when the ship was in the middle of retreating. Where the wind came from and how strong it was are examined in this study, based on synoptic (hundreds to thousands of kilometers) to small-scale (<2 km) weather features as well as information obtained from an extensive damage survey, in which drones were used for the first time in the meteorological damage survey history of China.

## Mesoscale systems that produced the strong wind: conventional observations

**Fig. 2:**
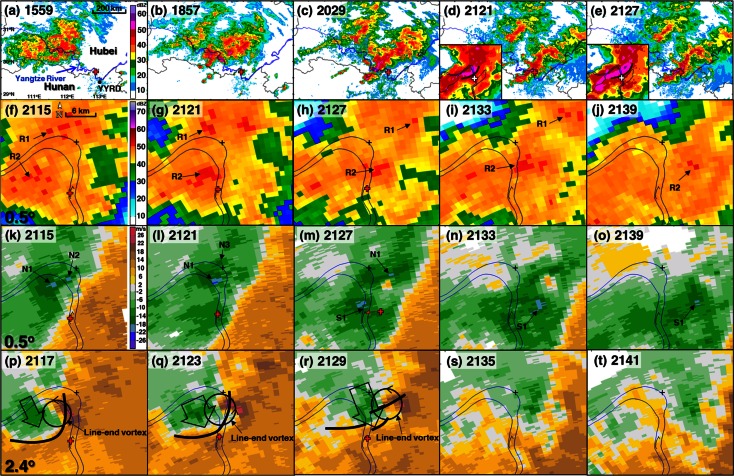
Evolution of radar observations from the Yueyang S-band Doppler radar (YYRD) (its location is denoted by the black dot in (**a**)). **a**–**e** Composite reflectivity over a large area. All other panels show the radar base reflectivity and radial velocity around the shipwreck location (red cross). The bow echo part in (**d**, **e**) is enlarged in their respective insets, with the leading edge of the 50-dBZ band in terms of composite reflectivity denoted by a thick black line and the shipwreck location denoted by a white cross. **f**–**j** Base reflectivity at the 0.5° elevation angle. **k**–**o** Radial velocity at the 0.5° elevation angle. **p**–**t** Radial velocity at the 2.4° elevation angle. The red crosses in some panels denote the location of Oriental Star at the corresponding time. In (**f**–**t**), the boundaries of the Yangtze River and the sailing trace of Oriental Star are depicted by blue and black lines, respectively. Heavy rainfall centers are denoted by R1 and R2 in (**f**–**j**). N1–N3 in (**k**–**m**) denote the locations of the radial velocity maximum in the area of the northern strong wind band. S1 in (**m**–**o**) shows the location of the radial velocity maximum in the area of the southern strong wind band. In (**p**–**r**), the line-end vortex is denoted by a black circle. The thick black lines in (**p**–**r**) denote the leading edge of the bow echo (in 50 dBZ) in terms of the composite reflectivity from the YYRD at the corresponding times. In (**k**–**t**), cool coloring indicates the inbound radial velocity toward the southeast to the Yueyang radar and the warm coloring indicates the outbound radial velocity toward the northwest away from the Yueyang radar

The ship ran into a squall line that night, as shown by the radar composite reflectivity^1^ images (Fig. 2a–e). A squall line is *a line of active thunderstorms, either contiguous or with breaks, including the contiguous precipitation area resulting from the existence of the thunderstorms* [1]. It is a type of mesoscale convective system (MCS) with a large length-to-width ratio that usually causes heavy rainfall, strong winds, hail, gustnadoes, and even tornadoes. An MCS is a *cloud system that occurs in connection with an ensemble of thunderstorms and produces a contiguous precipitation area on the order of 100* *km or more in horizontal scale in at least one direction* [1]. A tornado is *a rotating column of air, in contact with the surface, pendant from a cumuliform cloud, and often visible as a funnel cloud and/or circulating debris/dust at the ground* [1]. A gustnado is *a short*-*lived, shallow, generally weak, vertically oriented vortex found along a gust front* [1]. *Gustnadoes do not connect with any cloud*-*base rotation and are not tornadoes* (Available online at http://www.nws.noaa.gov/glossary/index.php?letter=g). MCSs are a very common type of severe convective weather system in China in the warm season, especially in central eastern China [2, 3].

**Fig. 3:**
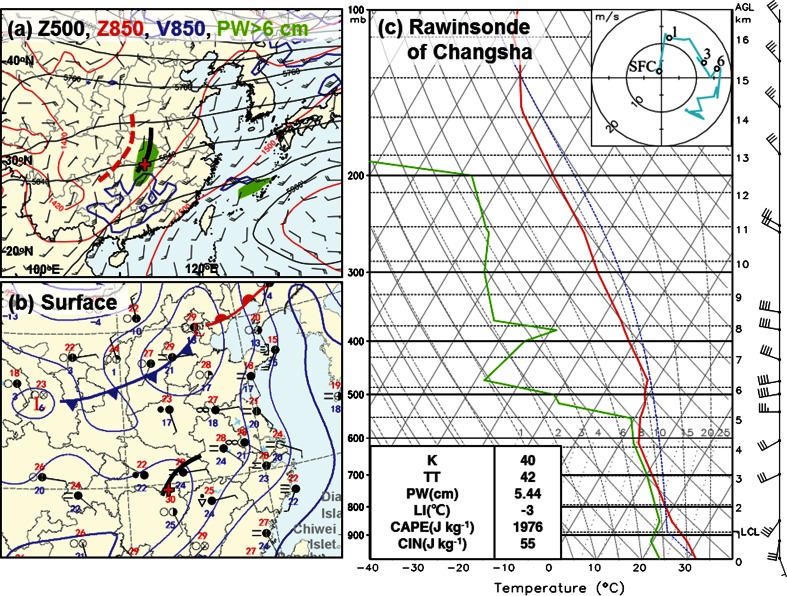
Synoptic weather map and rawinsonde observations from Changsha at 2000 LST June 1, 2015. **a** Geopotential height at 500 hPa (black contours; units: gpm) and 850 hPa (red contours; units: gpm) horizontal wind vectors at 850 hPa (the half barb, full barb, and flag denote 2, 4, and 20 m s^−1^, respectively) with the 850-hPa wind speed of 12 m s^−1^ plotted in blue contours. Areas with column-integrated precipitable water (PW) >6 cm are shown with green shading. The thick solid (dashed) arc shows the trough at 500 hPa (horizontal wind shear line at 850 hPa). The wreck location of Oriental Star is denoted by a red cross. **b** Surface synoptic map provided by the China Meteorological Administration with isobars (blue contours), weather station information (including temperature, dew point, wind, weather phenomenon, and cloud cover), lows (by “L”), and fronts. **c** Skew-T diagram and hodograph (inset) of the Changsha rawinsonde. Levels of surface, 1 km, 3 km, and 6 km are denoted by SFC, 1, 3, and 6 in the hodograph. The sounding parameters of K index, total totals (TT) index, PW, lifted index (LI), CAPE, and convective inhibition (CIN) are given in the bottom-left of the panel. LI, CAPE, and CIN are all surface-based values. The height of the lifting condensation level (LCL) is given in the bottom-right of the panel. K, TT, and LI are environmental stability indices. The K index is a measure of thunderstorm potential based on the temperature lapse rate, moisture content of the lower troposphere, and the vertical extent of the moist layer [1]. It is defined as the difference between temperature at 850 hPa and temperature at 500 hPa, plus the dew point temperature at 850 hPa, and then minus the difference between the temperature at 700 hPa and the dew point temperature at 700 hPa. The TT index is defined as the sum of two indices: vertical totals (VT) and cross totals (CT). VT is the difference between the temperature at 850 hPa and the temperature at 500 hPa. CT is the difference between the dew point temperature at 850 hPa and the temperature at 500 hPa [1]. LI is defined as the difference in temperature of a parcel lifted to 500 hPa (dry adiabatically to saturation and moist adiabatically above that) and the temperature at 500 hPa [1]

In the late afternoon on June 1, 2015, several convective cells developed within a cloud cluster near the boundary between Hubei and Hunan Provinces (Fig. 2a, b). Later on, with the development of a 500-hPa short-wave disturbance (the thick black solid line in Fig. 3a), an 850-hPa horizontal wind shear line (the thick red dashed line in Fig. 3a), an 850-hPa low-level jet (blue contours in Fig. 3a) providing rich moisture (green shading in Fig. 3a), and an inverted trough at the surface (the thick solid line in Fig. 3b), the scattered convective cells started to line up at around 1900 LST and merged into a squall line at 2006 LST. The band of 40-dBZ radar composite reflectivity had a width of about 30 km and a length of more than 200 km with a southwest–northeast orientation (Fig. 2c–e). After formation, the squall line moved to the east at a speed of ~40 km h^−1^. The rawinsonde at Changsha at 2000 LST showed a surface-based convective available potential energy (CAPE) of 1,976 J kg^−1^ and 0–6 km vertical wind shear of ~15 m s^−1^ with a veering hodograph (Fig. 3c and its inset), suggesting an environment favorable for the maintenance of the squall line. At 2115 LST, a bow echo formed in the middle of the squall line and passed over the wreck location during 2121–2130 LST (Fig. 2d, e). A bow echo is a *bow*-*shaped line of convective cells that is often associated with swaths of damaging straight*-*line winds and small tornadoes* [1]. Strong surface winds usually occur near the apex of a bow echo. Straight-line wind is a *current of air in which the ground*-*relative motion does not have any significant curvature* (used for distinction from winds in tornadoes, which have significant curvature) [1].

Due to the sparse distribution of surface weather stations (black dots in Fig. 1a; the nearest surface weather station, at Jianli, was about 13 km from the wreck location), no direct wind observation was available near the wreck location. Since the wind field in a squall line may change dramatically within several minutes and hundreds of meters, the wind observation at Jianli station could not represent what happened at the wreck location. One possible way of estimating the wind speed is through radar radial velocity analyses. Radar radial velocity data are available every 6 min with a radial resolution of 250 m and are thus very helpful for severe storm analyses.

## Wind estimation at ~700 m above ground level: radar analyses

The nearest radar from the wreck location was the operational S-band Doppler radar at Yueyang (YYRD in Fig. 2a), which was about 49 km from the wreck location. Although the radial velocity (*V*_*r*_) observation at the lowest elevation angle (0.5°) was about 700 m above ground level (AGL) at this distance, this was the only possible meteorological observation that could be used for wind estimation in this case. In the radar radial velocity field, the leading edge of a squall line is usually manifested as a boundary of warm and cool color representing outbound (positive) and inbound (negative) wind components in the radial direction with regard to the radar site, respectively, corresponding to its convergent wind. In this case, the radial velocity shifted from southeast to northwest at the leading edge of the squall line (Fig. 2k–o).

At 2115 LST, the ship crossed the leading edge of the squall line, moving from an area with largely southerly wind to an area with largely northerly wind (Fig. 2k). At this time, two maximum heavy rainfall centers, R1 and R2, were observed (Fig. 2f). Two maximum inbound *V*_*r*_ centers, N1 and N2 (Fig. 2k), reached 18–22 m s^−1^ about 6 km to the north of the wreck location, likely associated with an aloft rear-inflow jet^2^ (large open arrow in Fig. 2p) near the apex of the bow echo (thick black line in Fig. 2p, in terms of the leading edge of the 50-dBZ composite reflectivity band). One cyclonic line-end vortex on the northern end of the bow echo was evident. *Line-end (or book-end) vortices are mesoscale vortices observed at the ends of a line segment of convective cells, usually cyclonic on the northern end of the system and anticyclonic on the southern end, for an environment of westerly vertical wind shear (in the Northern Hemisphere). The vortices are generally strongest between 2 and 4 km AGL, but may extend from near the surface to about 8 km AGL. They have been observed at scales between 10 and 200 km, and often have lifetimes of several hours* [1].

At 2121 LST, the maximum inbound velocity center N1 shifted to the riverside and intensified to 22–26 m s^−1^ (Fig. 2l) with the strengthening (Fig. 2q) of the line-end vortex and rear-inflow jet. A maximum inbound velocity center N3 appeared about 5 km to the north of N1 (Fig. 2l), likely due to the downdraft associated with the enhanced rainfall of R1 (Fig. 2g). In the meantime, the rainfall of R2 shifted to the riverside about 2 km to the north of the ship’s northernmost location and significantly intensified. The radial velocity to the south of rainfall R2 intensified, possibly due to the downdrafts associated with R2 (Fig. 2g, l) and/or the rear-inflow jet near the apex of the bow echo. This was when the ship experienced the first strong wind (2123 LST).

At 2127 LST, the strong wind patch N1 shifted to about 3 km to the east of the Yangtze River and weakened with only two pixels maintaining an inbound *V*_*r*_ of 18–22 m s^−1^ (Fig. 2m), likely associated with the weakening of the line-end vortex and the rear-inflow jet (Fig. 2q, r). The bow echo broke into three linear pieces. The *V*_*r*_ (S1 in Fig. 2m) under and to the south of the rainfall R2 strengthened to 18–22 m s^−1^ about 1 km to the north of the shipwreck location. Strong surface winds may have occurred near the shipwreck location, likely due to the downdraft associated with the enhanced rainfall of R2 from about 2121 LST and/or the rear-inflow jet near the apex of the bow echo. The maximum rainfall shifted from the west to the east side of the Yangtze River and reduced in area (Fig. 2h). Thereafter, the heavy rainfall region shifted farther eastward and weakened, with corresponding weakening and shrinking *V*_*r*_ patches of 18–22 m s^−1^ to the south of the rainfall regions (Fig. 2i, j, n, o).

The above radar observation analyses suggest that there may have been two zonally oriented strong wind bands at the surface near the apex of the bow echo, corresponding to the two observed strong *V*_*r*_ bands at ~700 m AGL: One was about 6 km to the north of the wreck location and the other was directly over the wreck location. They were probably both related to enhanced local heavy rainfall under the aloft rear-inflow jet. Since there were no surface observations within about 13 km from the wreck location, an on-site survey was necessary to estimate the strength of surface winds by examining the damage to trees, buildings, utility poles, or other damage indicators in the disaster area. It is important to note here that all of the damage described in the next section happened during the passing of the squall line, according to the evolution depicted by the radar images, and was assumed to have been caused by a one-time passing of a single small-scale strong wind event embedded in the squall line.

## Surface wind estimation: on-site damage survey

When direct observations are unavailable, the most effective and reliable way to reveal the wind conditions near the ground is through a damage survey [4, 5]. Accordingly, in the present study, an extensive damage survey was conducted in the disaster area on both sides of the Yangtze River on two separate occasions: 2–3 June and 10–13 June. The routes of the damage surveys are shown in Fig. 1a by the black lines. The position, type, direction, diameter, and damage of all accessible fallen trees were recorded using rulers, cameras, compasses, and GPSs (Fig. 1c). The areas represented by the four red boxes and one white box in Fig. 1a were explored using two drones through both photography and video footage (Fig. 1d, e).

It was found that the damage caused by the passing of the bow echo was mainly in the form of snapped or uprooted trees. Although bent corns and slightly peeled roofs were also observed, they were highly infrequent and hard to rate, so the wind speed was estimated using the damaged trees only and according to the Enhanced Fujita (EF) scale [6, 7]. Likely due to the uncertainty in soil properties and the size of the trunks and crowns of trees, which may affect the degree of damage in a complicated way, making it hard to assess, the EF-scale method only distinguishes hardwood and softwood trees that experience five degrees of damage (DoD), namely *small limbs broken, large branches broken, trees uprooted, trunks snapped, and trees debarked with only stubs of largest branches remaining* [6]. The ranges of three-second wind speed estimation for snapped and uprooted hardwood trees are 42–60 and 34–53 m s^−1^, respectively. Here, as in our previously published paper [8], we conservatively assigned the expected values of 49 and 40 m s^−1^ to snapped and uprooted hardwood trees, respectively, both of which are within the range of EF1 (38–49 m s^−1^). It is important to note that the wind speed estimated using the EF-scale may have an error bar of ~18 m s^−1^ [9].

**Fig. 4:**
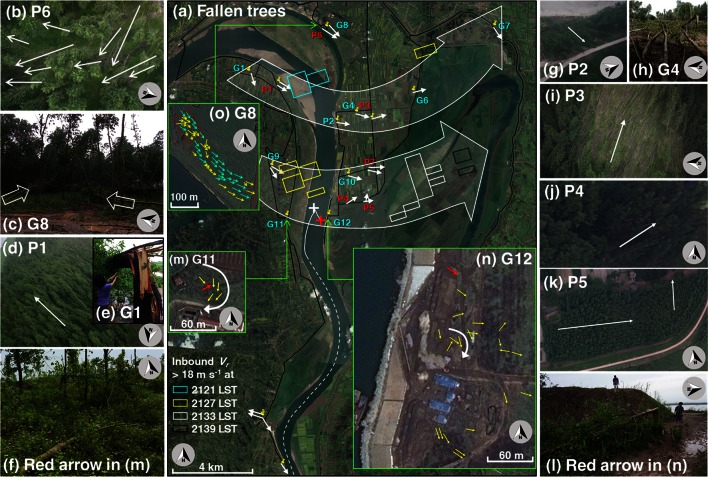
Information on fallen trees from both ground and aerial damage surveys. **a** An overall view of the fallen trees (thin white arrows) and the areas of inbound *V*_*r*_ > 18 m s^−1^ at the 0.5° elevation angle at 2121 (cyan), 2127 (yellow), 2133 (white), and 2139 (black) LST, respectively, while here only the inbound (negative) *V*_*r*_ reached such magnitude at these times corresponding to Fig. 2l–o. The two major wind bands are denoted by large white arrows. P1–7 denote the locations in aerial survey areas. All other information is as in Fig. 1a. The detailed distributions of downed (yellow arrows) or bent (cyan arrows) trees at G11, G12, and G8 are given in (**m**–**o**). The white arrows in (**m**, **n**) denote the curved wind pattern indicated by the fallen trees. **b**–**l** Pictures at a number of surveyed sites, as denoted in their respective captions. **b**, **d**, **g**, **i**–**k** were obtained from the drone surveys, while the pictures in (**c**, **e**, **f**, **h**, **l**) were taken by the authors on the ground. The picture in (**b**) covers the area denoted by the red box in (**o**). The pictures of the trees denoted by the red arrows in (**m**, **n**) are given in (**f**, **l**), respectively. North is indicated by an arrow in a gray-shaded circle in all panels

By plotting all the fallen trees on satellite images (available on Google Earth), two banded zones of apparent wind damage were recognized (Fig. 4a), with more severe damage observed in the northern zone than in the southern zone, which was consistent with the distribution of *V*_*r*_ (Fig. 2l, m; clusters of small colored boxes in Fig. 4a represent inbound *V*_*r*_ > 18 m s^−1^, corresponding to Fig. 2l–o). Considerable downburst straight-line and whirlwind damage was observed along or near the apex of the bow echo. A downburst is *an area of strong, often damaging, windsproduced by one or more convective downdrafts. Downbursts over horizontal spatial scales* ≤*4* *km are referred to as microbursts, whereas larger events with horizontal spatial scales* >*4* *km are termed macrobursts* [1]. Microbursts are capable of producing damaging straight-line winds of more than 45 m s^−1^, with peak winds that last 2–5 min.

A narrow swath of uprooted or snapped trees (yellow arrows in Fig. 4o) was observed within a wider swath of permanently bent trees (cyan arrows in Fig. 4o) of 400 × 50 m^2^ toward the east–southeast at Laotai wharf (G8 and P6 in Fig. 4a, o, b, c), with some slightly convergent and divergent patterns ~20 m wide (Fig. 4o, c), suggesting the happenstance of a microburst (near N3 in Fig. 2l). The damage was rated EF1, with a wind estimation of about 49 m s^−1^, based on the snapped trees [6, 8].

Slightly to the southwest, another microburst was suggested in a 1,200 × 300 m^2^ area of permanently bent trees to the southeast, with isolated snapped or uprooted trees along the Yangtze River to the south of Shunxing village (P1 in Fig. 4a, d). An aspen tree with a diameter of 50 cm was snapped in Shunxing village (G1 in Fig. 4a, e), corresponding to an estimated wind speed of about 49 m s^−1^. Southeastward across the river, two strong microbursts were indicated by downed trees in an area of 80 × 40 m^2^ (P2 in Fig. 4a, g) and 200 × 70 m^2^ (G4 and P3 in Fig. 4a, h, i; the aerial video of the damaged trees at this place is available in the supplementary material: video.mpg) in Xinzhou village. Permanently bent trees in an area of about 200 × 150 m^2^, with isolated snapped and uprooted trees, were found farther east at G6 (Fig. 4a), indicating another microburst. A tree with a diameter of 71 cm was uprooted and fell to the east–northeast at G7 in Fig. 4a, which was rated as EF1 with an estimated wind speed of about 40 m s^−1^.

The southern strong wind band was more closely related to the shipwreck. Isolated patches of fallen trees were found on both sides of the river. A microburst caused diverging fallen trees toward the southeast about 3 km to the northwest of the wreck location on the west side of the river (G9 in Fig. 4a). On the east side, several more microbursts were indicated by patches of trees bent in a similar direction with isolated snapped or uprooted trees (G10, P4, P5, and P7 in Fig. 4a, j, k).

Most importantly, damaged trees were observed on both sides of the Yangtze River immediately across the shipwreck location (G11 and G12 in Fig. 4a), collocating with the strong inbound radial velocity regions observed by the radar at 2127 LST (yellow box clusters in Fig. 4a). The physical nature of the fallen trees nearest to the wreck location on both shores of the river (G11 and G12 in Fig. 4a, m, n) was more complicated than that slightly more to the north or farther away. Specifically, the trees did not fall to the same direction, but instead showed a curved pattern ~30 m wide (Fig. 4m, n), within trees that permanently bent or fell to the southeast. This curved feature suggests the occurrence of small vortices on the flanks of surging outflow currents or microbursts (near S1 in Fig. 2m) near the heavy rainfall (R2 in Fig. 2g) and the apex of the bow echo (Fig. 2q, r, m). These small vortices were not the mesovortices observed by the radar at the 2.4° elevation angle. They were much smaller in size, shorter-lived, and might well have been confined to very low levels, likely caused by horizontal shear or turbulence associated with the downburst. This would have made it difficult for the S-band Doppler radar in this case to capture them. They were unlikely to have been tornadoes, because a tornado needs to possess rotating winds on the ground that connect to a cloud-base rotation under a cumuliform cloud [1]. From a damage point of view, a tornado usually causes a narrow damage swath with swath-scale convergent or curved debris [10]. It is unknown whether such vortices during the heavy rainfall that night connected to a cloud-base rotation. The damaged trees did not show a narrow swath. The areas with curved fallen trees were very small and localized. These vortices were unlikely to have been gustnadoes either, because they occurred in the outflow region far behind the gust front^3^ (not shown). Thus, they were more likely small vortices embedded in a microburst, rather than tornadoes or gustnadoes.

Damaged trees were used to estimate the wind speed near the shipwreck location. Isolated snapped or uprooted aspens with diameters of 15–20 cm were observed: The uprooted tree in Fig. 4f corresponds to the red arrow in Fig. 4m, which was about 1.6 km west of the wreck location and the snapped tree in Fig. 4l corresponds to the red arrow in Fig. 4n, which was about 0.6 km east of the shipwreck location. These instances of damage were caused by estimated winds of 49 m s^−1^, using the EF-scale, which is the expected value in the range of wind estimation for snapped hardwood trees (42–60 m s^−1^). The tree damage at these two locations (G11 and G12) was also estimated using the T-scale, commonly used in Europe (e.g., [11]), yielding a wind range result of 42–51 m s^−1^ (T3), similar to the result of the EF estimation. Considering the continuity of strong wind bands, as indicated by the *V*_*r*_ and less friction over the river, Oriental Star was likely hit by microburst straight-line wind and/or embedded small vortices with a wind speed of 49–60 m s^−1^, or at least 31 m s^−1^, considering an uncertainty of about 18 m s^−1^ in the estimation method [9], when it capsized.

## Summary and discussion

Hit by a severe thunderstorm, Oriental Star capsized in Jianli County, Hubei Province, at about 2131 LST on June 1, 2015, when it was sailing to Chongqing from Nanjing along the Yangtze River. A total of 442 lives were taken, among 454 passengers and crewmembers. No direct wind observations were available due to sparse surface observation stations. The present reported work attempted to reveal the weather phenomena during the event and estimated the wind speed at the wreck location, through radar analyses as well as both ground and aerial damage surveys. The results show that the cruise ship capsized when it encountered strong winds of at least 31 m s^−1^ near the apex of a bow echo embedded in a squall line. The damage surveys demonstrate that such strong winds were likely caused by microburst straight-line wind and/or embedded small vortices.

No adequate observational evidence of a tornado or gustnado was found in this event. Determining the occurrence of a tornado can be a difficult question in some situations. When heavy rainfall takes place, it is almost impossible to observe a tornado due to limited visibility, especially at night. According to the definition of a tornado by the Glossary of Meteorology [1], three necessary conditions need to be satisfied for a vortex to be considered as a tornado: (1) rotating winds on the ground; (2) connection with a cloud-base rotation; and (3) location under a cumuliform cloud. From the damage point of view, a tornado usually causes a narrow damage swath with a storm-scale curved or convergent debris. Gustnadoes are often mistaken for tornadoes due to difficulties in assessing condition (2) or ignorance of conditions (2) and/or (3). A small vortex that satisfies condition (1) and (3) but not (2) should be considered a gustnado if it forms right along the gust front. In this event, conditions (1) and (3) were met around the shipwreck location, but whether or not condition (2) was met remains unknown. Considering that the areas with convergent or curved trees were quite small and localized, without showing a narrow swath with a swath-scale convergent or curved debris [10], and they did not appear along the gust front, they more likely resulted from small vortices forming on the flanks of surging outflow currents or microbursts, rather than tornadoes or gustnadoes.

## Electronic supplementary material

Supplementary material 1 (MPG 31774 kb)

## Footnotes

1 Composite reflectivity: *A display**or mapping of the maximum radar reflectivity factor**at any altitude as a function of position on the ground* [1]. Large composite reflectivity usually suggests heavier rainfall.

2 Rear-inflow jet: *A mesoscale circulation feature in which a system-relative current of air enters and flows through the stratiform precipitation region of mesoscale convective systems from the rear. The rear-inflow jet supplies potentially cold and dry midlevel air that aids in the production of convective and system-scale downdrafts* [1].

3 Gust front: *The leading edge of a mesoscale pressure dome separating the outflow air in a convective storm from the environmental air* [1].
